# Proteomics Reveals Mechanisms of Metabolic Dysregulation in Soman Neurotoxicity

**DOI:** 10.3390/toxics13090766

**Published:** 2025-09-10

**Authors:** Xing-Xing Zong, Qian Jin, Tong Shi, Ruihua Zhang, Jingjing Shi, Chen Wang, Liqin Li

**Affiliations:** State Key Labroratory of Chemistry for NBC Hazards Protection, Beijing 102205, China; jxjjzong@163.com (X.-X.Z.); jinqian160@163.com (Q.J.); tong198282@126.com (T.S.); 15201517118@163.com (R.Z.); shijingjing9287@126.com (J.S.)

**Keywords:** soman, organophosphate, neurotoxicity, hippocampus, metabolic dysregulation, proteomics

## Abstract

Soman, an organophosphorus nerve agent, induces neurotoxicity primarily by inhibiting acetylcholinesterase, triggering a series of pathological events including cholinergic crisis, seizures, calcium overload, oxidative stress, mitochondrial dysfunction, and neuronal death. Nevertheless, the mechanisms underlying metabolic dysregulation—especially after repeated exposure—remain poorly characterized. To address this, we used SWATH-based proteomics to analyze changes in the hippocampal proteome following a repeated soman exposure regimen in a model of hippocampal injury. We identified 38 differentially expressed proteins, predominantly enriched in metabolic pathways. KEGG annotation indicated that these were mainly involved in carbohydrate, amino acid, and lipid metabolism, with specific roles in calcium signaling, tryptophan and tyrosine metabolism, alanine, aspartate and glutamate metabolism, and glyoxylate and dicarboxylate metabolism. Overall, our results demonstrate significant disruption of key metabolic pathways, particularly affecting carbohydrate and amino acid metabolism. We suggest that soman-induced hippocampal damage arises not only from acute calcium overload but also from persistent metabolic dysregulation that impairs energy production and biosynthetic processes. All of our preliminary results shed light on the nature of the biological process and target in the metabolism and provide basic research for the treatment, diagnosis, and prevention of nerve-agent-induced brain damage.

## 1. Introduction

Organophosphorus nerve agents (OPNAs) are well-known chemical agents, primarily comprising tabun, soman, sarin, cyclosarin, and VX. Among these, tabun, sarin, and VX have been used multiple times in recent decades. In April 1997, the Chemical Weapons Convention came into force, prohibiting the development, production, stockpiling, and use of chemical weapons. This significant milestone has reduced the likelihood of large-scale military use of chemical agents. However, the use of nerve agents by terrorist organizations in Japan, as well as recent events in the Middle East, demonstrates the growing interest of non-state actors in acquiring chemical agents and using these toxic substances to attack civilians [1]. Consequently, we continue to face the risk of accidental or deliberate poisoning by nerve agents, as well as the potential threat of terrorist use of these agents.

OPNAs can irreversibly inhibit acetylcholinesterase (AChE) because the AChE–OPNA conjugate undergoes rapid dealkylation in a process termed “aging”. This results in the accumulation of high levels of the neurotransmitter acetylcholine (ACh) in the synaptic cleft [2]. The accumulation of ACh induces various pathological effects, such as respiratory depression, seizures, and status epilepticus. Respiration is regulated by the respiratory center in the brainstem, which comprises a cluster of neurons possessing muscarinic and nicotinic receptors. Excess ACh can modulate the activity of these receptors, leading to central apnea [3,4,5]. While mortality due to respiratory failure following organophosphate poisoning is primarily mediated through central mechanisms, the peripheral muscarinic effects resulting from acetylcholinesterase inhibition—manifested as a peripheral cholinergic crisis—can significantly exacerbate respiratory compromise [6]. On the other hand, McDonough and Shih proposed a three-phase model that elucidates the pathophysiological progression from nerve-agent-induced seizures to severe status epilepticus [7]. Repeated seizure-induced depolarization and prolonged stimulation of NMDA receptors, ultimately resulting in excessive calcium influx, are thought to be the primary causes of neuropathy [8] These pathophysiological processes, which constitute the primary events in neurotoxicant-induced neurological damage, have been characterized through the application of global molecular techniques [9].

The excessive calcium influx triggers downstream molecular events, including the activation of protein kinases, catabolic enzymes, and free radical generation [10,11]. Additionally, several downstream signaling pathways, such as calcium signaling, the protein kinase C pathway, MAP kinases, and oxidative signaling, are activated by CaMKII. These pathways lead to neuropathology (inflammation) and associated dysfunction (cytoskeletal and mitochondrial damage) through complex and diverse mechanisms [12].

Soman is an organophosphorus nerve agent that is particularly susceptible to “aging” compared to other OPNAs [13]. Therefore, understanding the mechanism of soman poisoning and developing effective antidotes is crucial for protecting public health and security. However, the biochemical and metabolic mechanisms involved in soman-induced brain damage are not yet fully understood. CaE was the primary enzyme responsible for the degradation metabolism of soman in the body. Primates, including humans, have almost no CaE, while guinea pigs have low levels, and rats and mice have high levels of plasma and tissue CaE [14]. This effectively explains why guinea pigs were more sensitive to the toxicity of soman [15]. In the present study, soman was selected to investigate the proteomic changes in metabolism caused by nerve-agent-induced hippocampal damage using SWATH-based proteomic technology.

## 2. Materials and Methods

### 2.1. Chemicals and Reagents

Soman (98% purity) was obtained from the China Analytical Chemistry Laboratory (Beijing, China). Ammonium acetate, iodoacetamide, and triethylammonium bicarbonate (TEAB) buffer was obtained from Sigma-Aldrich (St. Louis, MO, USA). Acetone (analytical grade) and sodium dodecyl sulfate (SDS) were purchased from Sinopharm Chemical Reagent Co., Ltd. (Shanghai, China). Sequencing-grade modified trypsin was acquired from Promega (Shanghai, China). Methanol, acetonitrile, formic acid, protein ladder, protease inhibitor cocktail, and Bond-Breaker™ TCEP solution were procured from Thermo Fisher Scientific (Shanghai, China). Deionized water (18.2 MΩ·cm) was purified using a Milli-Q water purification system (Millipore, Bedford, MA, USA). Fourteen standard compounds of amino acids and related metabolites were supplied by Shanghai Macklin Biochemical Co., Ltd. (Shanghai, China). and the specific analyte information is provided in Table S6. All other chemicals and solvents were of analytical grade and were commercially purchased from Aladdin Chemical Reagent Co., Ltd. (Beijing, China).

### 2.2. Animals and Drug Administration

Hartley guinea pigs (seven weeks old, male, 280–320 g) were purchased from Beijing Huafu Kang Biotechnology Co., Ltd. (Beijing, China). The guinea pigs were housed with access to tap water and fed a certified standard diet. Humidity and temperature were controlled at 40–70% and 20–22 °C with a 12 h light/dark cycle, respectively, as extreme temperature and humidity can predispose them to health problems [16]. All feeding and experimental procedures were conducted in accordance with the guidelines of the State Key Laboratory of NBC Protection for Civilian.

One week following the arrival of the animals, half of the guinea pigs were randomly assigned to undergo environmental enrichment for a period of one week. This intervention was administered twice daily, with each session lasting 60 min. The remaining 24 animals were housed individually in standard cages. The enriched environments were equipped with a variety of cognitive and physical stimuli, including horizontal platforms, a small running wheel, tunnels, a wooden ladder, wooden blocks, glass balls, jars, a bridge, and a maze. To maintain novelty, the spatial configuration of these objects was altered on a daily basis. After one week of acclimatization, the guinea pigs were randomly divided into two groups (control and soman-treated groups, *n* = 10/group). Guinea pigs in the control group received normal saline, while those in the soman-treated group received a preliminary subcutaneous injection of 0.2 × LD_50_ soman (11.0 μg/kg). The injection was administered subcutaneously at a dose of 1.0 mL/kg of body weight between the shoulder blades. The injection was repeated daily for 14 days. The LD_50_ of soman was determined to be 55 μg/kg based on previous studies [17].

### 2.3. Morris Water Maze Experiment

The Morris water maze test, incorporating a probe trial, was employed to evaluate spatial memory performance in guinea pigs from both the control and soman-exposed groups (*n* = 6 for each group). The experimental protocol was adapted from a previously established method [18]. A circular black-painted pool with a diameter of 1.5 m was filled with water (30 cm depth, maintained at 26 ± 2 °C), and a hidden platform (9 cm in diameter) was placed in the southeast quadrant, submerged 2 cm below the water surface. A video camera mounted above the center of the pool and connected to a computerized tracking system (Techman Technology Co., Ltd., Chengdu, China) was used to record the swimming time, path length, and velocity for offline analysis.

At the start of each trial, animals were introduced into the pool facing the wall from one of four pseudorandomized starting locations (north, east, south, west). Each subject was allotted a maximum of 60 s to locate the submerged platform. Four trials per day were conducted over five consecutive days as part of the acquisition phase. On the sixth day, a probe trial was performed by removing the platform and allowing each animal to swim freely for 60 s. The time spent in each of the four quadrants was measured to assess spatial memory retention.

### 2.4. Histopathologic Study

On the 14th day following daily soman administration, guinea pigs from both the control and soman-treated groups (*n* = 3 per group) were anesthetized via inhalation of isoflurane and transcardially perfused with phosphate-buffered saline (PBS), followed by perfusion with 4% paraformaldehyde. Whole brains were subsequently extracted and subjected to fixation and dehydration. The dehydrated tissue samples were embedded in paraffin and coronally sectioned at a thickness of 5 µm. These sections were then stained with hematoxylin and eosin (H&E) for histological examination of the hippocampal structure. Morphological alterations in pyramidal neurons within the cornu ammonis 1 (CA1) region of the hippocampus were analyzed using light microscopy.

### 2.5. Hippocampal Tissue Sample Preparation

After the Morris water maze test, the guinea pigs from the control and soman-treated groups (*n* = 6 for each group) were anaesthetized by inhalation of isoflurane and transcardially perfused with phosphate-buffered saline (PBS). Finally, the tissues were minced and stored at −80 °C for ELISA assay, metabolite analysis, and proteomic analysis.

### 2.6. AChE Activity Assay

The hippocampal sample (approximately 50 mg, *n* = 4 for each group) was homogenized in 50 mM Tris-HCl buffer (pH 8.0) to obtain a 5% (*w*/*v*) homogenate. The AChE activity was measured by Ellman assay.

### 2.7. Enzyme-Linked Immunosorbent Assay

Frozen hippocampal tissues (approximately 50 mg, *n* = 6 for each group) were quickly thawed on ice and homogenized in 500 µL ice-cold 0.9% normal saline. After a 10 min centrifugation (10,000× *g*) at 4 °C, supernatants were employed to assess the concentrations of HYI and CAMK2. The HYI and CAMK2 (Jingmei Biotechnology Co., Ltd., Yancheng, China) were tested using an ELISA kit, according to the manufacturer’s instructions. Absorbance was recorded at 450 nm for each well, and the sample concentrations were determined based on a standard curve. The data were then subjected to statistical analysis.

### 2.8. Determination of Metabolites in Specific Amino Acid Metabolic Pathways

An appropriate amount of analyte standards was accurately weighed and individual stock solutions were prepared using methanol or water. Appropriate volumes of each stock solution were transferred to prepare a mixed standard solution. The mixed standard solution was diluted to the desired concentration with 10% formic acid in methanol–water (1:1, *v*/*v*) to obtain the working standard solution. Compound information and concentration levels are detailed in Table S7. Both stock and working standard solutions were stored at 0 °C.

Hippocampus samples (50 mg, *n* = 4 for each group) were homogenized in 0.8 mL of pre-cooled deionized water. The extraction solution was centrifuged at 12,000 rpm for 15 min at 4 °C and 0.2 mL of supernatant was transferred into a 2 mL EP tube. Exactly 400 μL of a solution containing 10% formic acid in methanol–water (1:1, *v*/*v*) was added. The mixture was vortex mixed for 30 s and then centrifuged at 12,000 rpm and 4 °C for 5 min. A total of 100 μL of the supernatant was collected. Exactly 100 μL of a 100 ng/mL isotope-labeled internal standard solution was added, followed by vortex mixing for 30 s. The supernatant was then filtered through a 0.22 μm membrane. The filtrate was transferred to an autosampler vial for low-concentration analyte analysis. On other hand, 20 μL of the original supernatant was collected and diluted with 380 μL of the 10% formic acid in methanol–water (1:1, *v*/*v*) solution. After vortex mixing for 30 s, 100 μL of this diluted sample was taken. Exactly 100 μL of a 100 ng/mL dual-isotope-labeled internal standard solution was added, followed by vortex mixing for 30 s. The mixture was then filtered through a 0.22 μm membrane, and the filtrate was transferred to an autosampler vial for high-concentration analyte analysis.

The separation of chromatographic conditions was performed on an ACQUITY UPLC^®^ BEH C18 column (2.1 × 100 mm, 1.7 μm; Waters Corp., Milford, MA, USA) maintained at 40 °C. The injection volume was 5 μL. Mobile phases consisted of 50% methanol in water containing 0.1% formic acid (A) and 10% methanol in water containing 0.1% formic acid (B). A gradient elution program was employed at a flow rate of 0.3 mL/min and performed with the following schedule: 10–30% of A at 0–6.5 min, 30–100% of A at 6.5–7.0 min, 100% of A at 7.0–14.0 min, 100–10% of A at 14.0–14.5 min, and 10% of A at 14.5–17.5 min. Mass spectrometry was conducted using an electrospray ionization (ESI) source in positive ion mode. The following parameters were used: ion source temperature, 500 °C; ion spray voltage, 5000 V; collision gas, 6 psi; curtain gas, 30 psi; nebulizing gas, 50 psi; and auxiliary gas, 50 psi. Detection was performed in multiple reaction monitoring (MRM) mode. The specific transitions used for quantification are provided in Table S8.

### 2.9. Proteomic Profile Analysis

Hippocampal tissues (100 μg; *n* = 4 for each group) were homogenized in protein lysis buffer (8 M urea, 1% SDS) and incubated on ice for 30 min. After centrifugation (12,000× *g*, 4 °C, 30 min), the supernatant was collected and the protein concentration was determined using the bicinchoninic acid (BCA) assay. The lysates were then reduced with 10 mM tris (2-carboxyethyl)phosphine (TCEP) at 37 °C for 60 min, followed by alkylation with iodoacetamide (IAM). After centrifugation (10,000× *g*, 4 °C, 20 min), the precipitate was collected and resuspended in 100 μL of TEAB to a final concentration of 100 mM. Trypsin was added at a 1:50 ratio of trypsin to protein mass and incubated overnight at 37 °C. An equal amount of trypsin-digested peptides was vacuum-dried and resuspended in UPLC sample preparation buffer (phase A: 5 mM ammonium hydroxide solution containing 2% acetonitrile, pH 10). The mixed peptides were fractionated using a Vanquish Flex binary ultra-high-performance liquid chromatography system ((Thermo Fisher Scientific, Inc., Waltham, MA, USA)) on an ACQUITY UPLC BEH C18 column (1.7 µm, 2.1 mm × 150 mm, Waters Corp., Milford, MA, USA) to increase proteome depth.

On the Q Exactive HF-X instrument (Thermo Fisher Scientific, Inc., Waltham, MA, USA), data-independent acquisition (DIA) was performed using automated switching between full MS and MS/MS scans. Full MS spectra were acquired within a mass range of *m*/*z* 350–1500 at a high resolution in the Orbitrap analyzer (Thermo Fisher Scientific, Inc., Waltham, MA, USA). Precursor ions were selectively transferred into the collision cell and fragmented via high-energy collisional dissociation (HCD). Data processing was carried out using Spectronaut (Biognosys AG, version 14) with dynamic iRT prediction enabled. Mass accuracy was ensured through extensive mass calibration. For protein quantification, up to six peptides per protein were selected, and three fragment ions per peptide were used for quantitative analysis. The following criteria were applied: protein FDR ≤ 0.01, peptide FDR ≤ 0.01, peptide confidence ≥ 99%, and XIC width ≤ 75 ppm, excluding shared and modified peptides. Peak areas were calculated, and quantitative results were obtained.

### 2.10. Statistical Analysis and Bioinformatics Analysis

Differences between the control and soman groups were determined by a one-way ANOVA followed by Tukey’s post-hoc test for multiple comparisons. All data were expressed as the mean ± SEM, and *p*-values below 0.05 were considered statistically significant. The statistical analyses were performed by GraphPad Prism 9.0.

Annotation analysis of differentially expressed proteins was performed based on Gene Ontology (GO) (http://geneontology.org/, accessed on 9 February 2024) for biological processes and the Kyoto Encyclopedia of Genes and Genomes (KEGG) (http://www.genome.jp/kegg/, accessed on 15 March 2024). Enrichment analyses were conducted using the KEGG, and the significance of pathways was determined based on a *p*-value of <0.05 using Fisher’s exact test. Each pathway contained at least two differentially expressed proteins. Statistical analyses were performed using one-way analysis of variance (ANOVA) followed by Tukey’s post-hoc test for differentially expressed proteins.

## 3. Results

### 3.1. Effects of GD on Spatial Learning and Memory in Guinea Pigs

**Figure 1:**
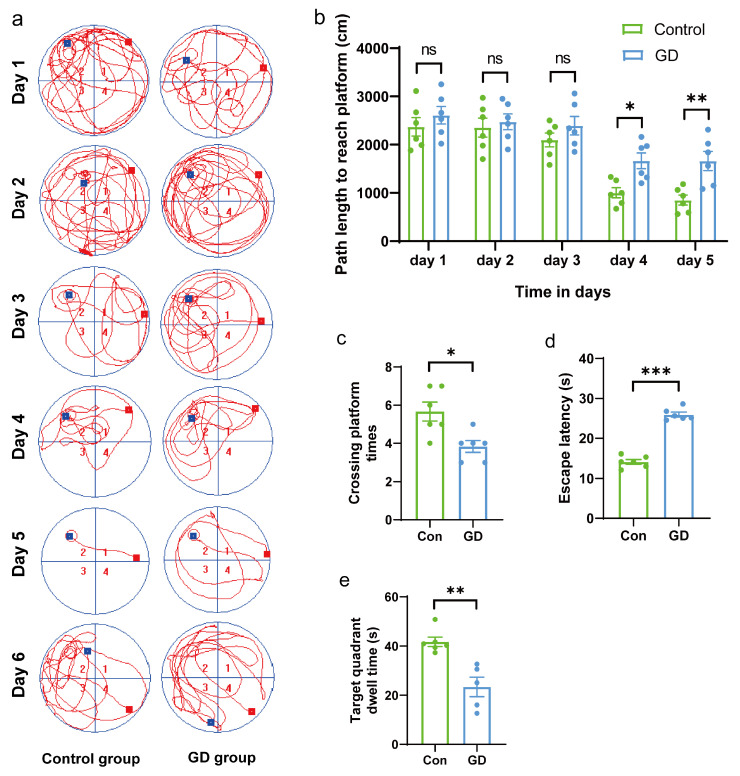
Results from the Morris water maze experiment. (**a**) Representative swimming trajectories in the Morris water maze experiment of each group. (**b**) Path length to reach platform of each group in training phase (day 1 to day 5). (**c**) The crossing platform times in the space exploration test. (**d**) The escape latency in the positioning navigation test. (**e**) The target quadrant dwell time in the space exploration test. The red squares represent drop points and green squares represent exit points. Data are presented as mean ± SEM; *n* = 6 for each treatment; ns indicated *p*-value > 0.05; * indicated *p*-value < 0.05, ** indicated *p*-value < 0.01, *** indicated *p*-value < 0.005 compared with control group. Data were analyzed using two-way analysis of variance and independent and paired-samples Student’s *t*-test.

In training trials, as shown in Figure 1b, the control group showed a progressive decrease in path length to reach the platform, indicating that training trials were completely effective in the control group. Meanwhile, the GD group showed a less progressive decrease in path length to reach the platform and had a significantly longer path length to reach the platform compared to the control group on day 4 and day 5 (day 4, *p* < 0.05; day 5, *p* < 0.01). Additionally, swimming trajectories on the 1th to 5th days revealed that those in the GD group were more disorganized and purposeless compared to those in the control group.

In the probe trial, the escape latency in the soman group was significantly longer (*p* < 0.05) compared to the control group in the Morris water maze task (25.95 ± 1.51 s vs. 10.29 ± 4.71 s; Figure 1d). The number of crossings through the platform position was also significantly reduced (*p* < 0.05) in the soman group (3.83 ± 0.75) compared to the control group (5.67 ± 1.21; Figure 1c). Analysis of the swimming trajectories on the 5th and 6th days revealed that the trajectories of the guinea pigs in the soman group were significantly more disorganized and purposeless compared to those in the control group (Figure 1a). During the probe trial, the percentage of time spent in the target quadrant was analyzed. The soman group spent significantly less time in the target quadrant (23.42 ± 8.80%) compared to the control group (41.74 ± 4.69%; Figure 1e). These results suggest that repeated exposure to soman impairs spatial learning and memory in guinea pigs.

### 3.2. Neuronal Changes in the Hippocampal Tissues of Guinea Pigs

**Figure 2:**
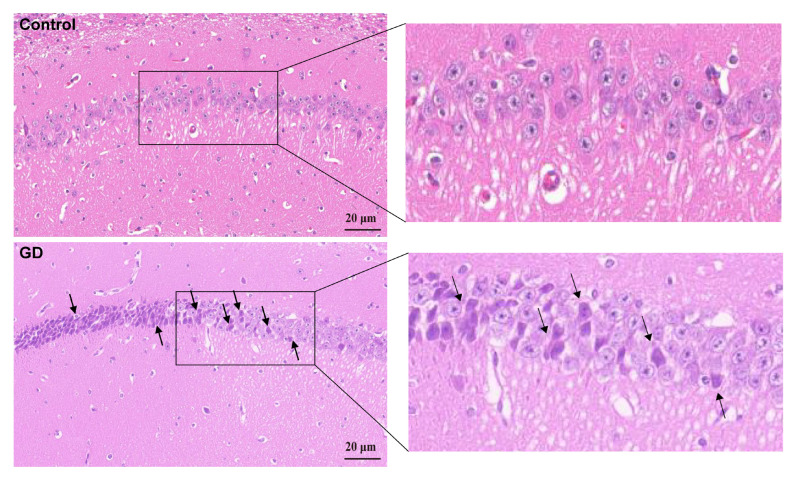
Neuronal pathological changes in the guinea pigs’ hippocampal CA1 region in soman group and control group. Black arrows indicate neuronal depletion in the hippocampal CA1 region was evident with disrupted cellular alignment in soman group.

Compared with the control group by HE staining, neuronal depletion in the hippocampal CA1 region was evident with disrupted cellular alignment in the GD group (Figure 2). This finding indicates significant structural pathology in guinea pig hippocampal tissues after repeated exposure to soman.

### 3.3. AChE Activity in Guinea Pig Hippocampal Tissues After Repeated Exposure to Soman

**Figure 3:**
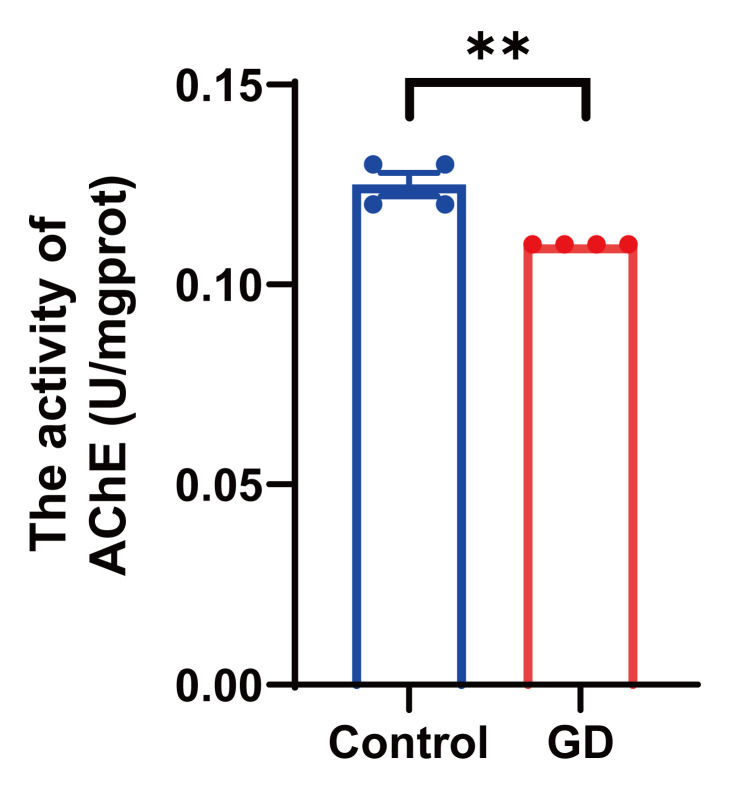
The measured results of AChE activity in the guinea pig hippocampus following soman exposure. Data are presented as mean ± SEM. *n* = 4 for each group; ** indicated *p*-value < 0.01 compared with control group. Data were analyzed using independent and paired-samples Student’s *t*-test.

AChE activity in the soman-treated group was determined to be 0.11 ± 0 U/mgprot and in the control group was determined to be 0.125 ± 0.002887 U/mgprot (Figure 3). The evidence suggests that hippocampal AChE function was potently suppressed in guinea pigs after soman exposure.

### 3.4. Hippocampal Proteomic Profiles

**Figure 4:**
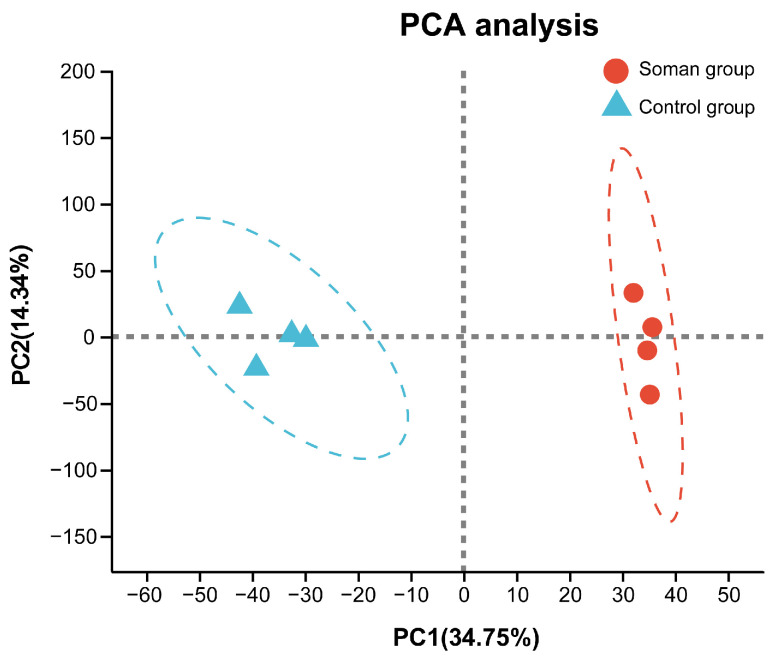
PCA analysis for differential protein from control group and soman group (*n* = 4 for each group).

In the hippocampus of guinea pigs, a total of 4523 proteins with one or more unique peptides were identified with a false discovery rate (FDR) of <1% and quantified using tandem mass tag (TMT) technology following soman exposure. Principal component analysis (PCA) were employed to analyze and compare samples from the soman and control groups. The model quality was assessed using R^2^X and Q^2^Y parameters. The PCA results (Figure 4) indicated that R^2^X = 34.75% and Q^2^Y = 14.34%, demonstrating significant differences between the control and soman groups, with minimal variation within each group.

### 3.5. Differential-Protein-Set Annotation Analysis

To characterize the differentially expressed proteins, Gene Ontology (GO) and Kyoto Encyclopedia of Genes and Genomes (KEGG) functional annotations were performed. GO functional annotation revealed that these proteins were primarily associated with a “cellular process” (78.4%), “biological regulation” (59.8%), a “metabolic process” (40.7%), a “developmental process” (28.6%), a “response to stimulus” (26.6%), “localization” (20.6%), a “multicellular organismal process” (15.1%), an “immune system process” (7.7%), and “locomotion” (7.5%) in the “biological process” (BP) category (Figure S1). KEGG functional annotation classified the differentially expressed proteins into categories such as “amino acid metabolism” (7.4%), “lipid metabolism” (4.4%), “carbohydrate metabolism” (7.9%), and “nucleotide metabolism” (3.0%) within the “metabolism” category (Figure S2).

Among the 38 differentially expressed proteins annotated in the “metabolism” category, a differential-expression protein set was constructed for further analysis and detailed information on these proteins is presented in Table S1. The top five differentially expressed proteins in this set were calcium/calmodulin-dependent protein kinase (CAMK2), tyrosine 3-hydroxylase (TH), sphingomyelin phosphodiesterase 3 (SMPD3), phosphoglycerate mutase (BPGM), and putative hydroxypyruvate isomerase (HYI). Detailed information on these proteins is presented in Table 1.

### 3.6. KEGG Functional Enrichment

**Figure 5:**
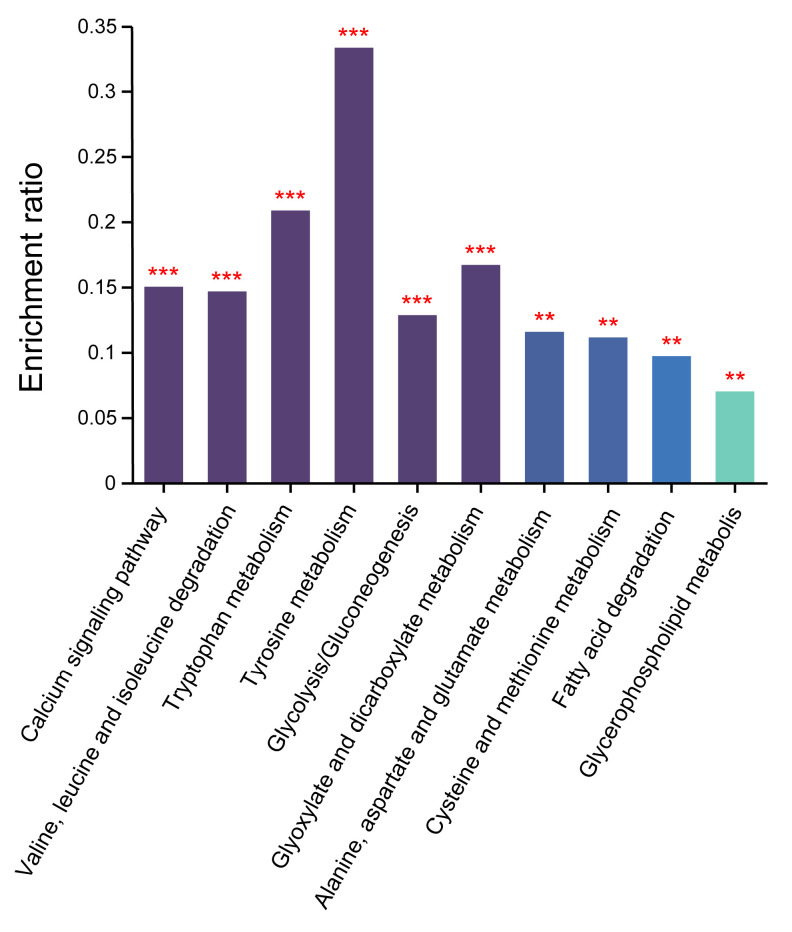
KEGG enrichment for differential protein in the guinea pigs hippocampus following soman exposure in category of “metabolism”. ** indicated *p*-value < 0.01, *** indicated *p*-value < 0.005 compared with control group.

These differentially expressed proteins were analyzed based on KEGG functional enrichment with an adjusted *p*-value < 0.05 by Fisher’s exact test. The results showed that these differentially expressed proteins were enriched in the calcium signal pathway; valine, leucine and isoleucine degradation; tryptophan metabolism; tyrosine metabolism; glycolysis/gluconeogenesis; glyoxylate and dicarboxylate metabolism; alanine, aspartate and glutamate metabolism; cysteine and methionine metabolism; glycerophospholipid metabolism; and fatty acid degradation (Figure 5).

#### 3.6.1. Calcium Signal Transduction and Transport

Calcium/calmodulin-dependent protein kinase (CAMK2), protein kinase C (PRKC), protein phosphatase 3 regulatory subunit (PPP3R), and calmodulin (CALM) were enriched in the calcium signaling pathway. These proteins were all upregulated, indicating that the calcium signaling pathway was activated. In addition, calbindin D28k (CB), calretinin (CR), and parvalbumin (PV) were downregulated. The specific information regarding this pathway is shown in Table S2.

#### 3.6.2. Amino Acid Metabolism

For the second category, “amino acid metabolism”, branched-chain amino acid aminotransferase (BCAT2), 3-hydroxyisobutyrate dehydrogenase (HIBADH), methylmalonyl-CoA isomerase (MMUT), and acetyl-CoA acetyltransferase 2 (ACAT2), which were enriched in valine, leucine, and isoleucine degradation, were found to be downregulated. ACAT2, aldehyde dehydrogenase (ALDH), and amine oxidase (MAOB) were enriched in tryptophan metabolism. GOT1 and malate dehydrogenase (MDH1), which were enriched in cysteine and methionine metabolism, were downregulated as well. GOT1, (S)-3-amino-2-methylpropionate transaminase (ABAT), and succinate-semialdehyde dehydrogenase (ALDH5A1), which were enriched in alanine, aspartate, and glutamate metabolism, were downregulated. GOT1, tyrosine 3-hydroxylase (TH), S-(hydroxymethyl) glutathione dehydrogenase (ADH5A1), and MAOB, which were enriched in the tyrosine metabolism, were downregulated, except for MAOB, which was upregulated. The specific information on this pathway is shown in Table S3.

#### 3.6.3. Lipid Metabolism

For the second category, “lipid metabolism”, diacylglycerol kinase (DGKG), monoglyceride lipase (MGLL), diacylglycerol kinase (DGKZ), sphingomyelin phosphodiesterase 3 (SMPD3), and lysophospholipase 2 (LYPLA2), which were enriched in the glycerophospholipid metabolism, were found to be upregulated. ACAT2 and ADH5, which were enriched in the fatty acid degradation, were downregulated. The specific information on this pathway is shown in Table S4.

#### 3.6.4. Carbohydrate Metabolism

For the second category, “carbohydrate metabolism”, differentially expressed proteins were mainly enriched in the “glycolysis/gluconeogenesis,” “TCA cycle,” and “glyoxylate and dicarboxylate metabolism” pathways. Specifically, phosphoglycerate kinase (PGK1), phosphoglycerate mutase (BPGM), phosphoglucomutase 1 (PGM1), and PGM2L1, which were enriched in the “glycolysis/gluconeogenesis” pathway, were found to be downregulated. Similarly, MDH1, ACAT2, and cytochrome C (CYC), which were enriched in the “TCA cycle,” were downregulated. In addition, MMUT, ACAT2, and putative hydroxypyruvate isomerase (HYI), which were involved in the glyoxylate and dicarboxylate metabolism, were downregulated. Specific information on this pathway is provided in Table S5.

### 3.7. Validation of Key Proteins by ELISA

**Figure 6:**
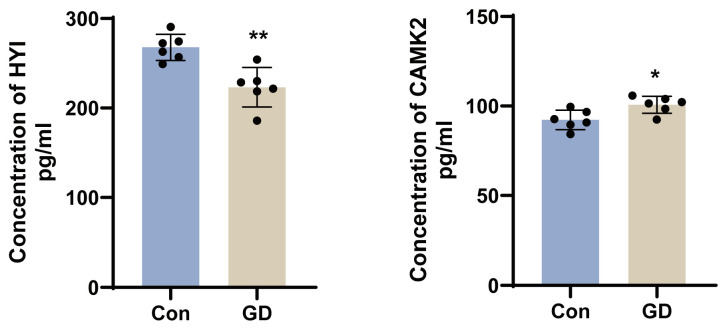
The results of HYI and CAMK2 levels determined by ELISA following soman exposure. Data are presented as mean ± SEM. *n* = 6 for each treatment; * indicated *p*-value < 0.05, ** indicated *p*-value < 0.01, compared with control group. Data were analyzed using two-way analysis of variance and independent and paired-samples Student’s *t*-test.

To validate the alterations of key differentially expressed proteins after subacute soman exposure, HYI and CAMK2 were representatively selected and subjected to ELISA (Figure 6). Hippocampus samples from six guinea pigs in the soman group and six guinea pigs in the control group were tested for protein expression. As a result, the abundance of CAMK2 increased (*p* < 0.05) in the hippocampus with 11.0 μg/kg of soman treatment. In addition, the abundance of HYI decreased (*p* < 0.01) in the hippocampus. These results were consistent with those obtained from LC–MS/MS.

### 3.8. Effect of Soman on Metabolite Levels in Specific Amino Acid Metabolic Pathways

The total ion chromatograms of those standards, samples, and blank matrix by UPHLC–MS/MS are shown in Figure S3. There was no interference peak observed in the blank matrix of the hippocampus. Quantification was performed within their respective linear ranges (typically R^2^ ≥ 0.99), as established during method validation (Table S9). The intra- and inter-day precision, expressed as relative standard deviations (RSDs), were both below 11.5%. Additionally, the accuracy for both intra- and inter-day analyses fell within ±12.6% for all analytes. Recoveries of quality control (QC) samples at three concentrations (low, medium, and high) ranged from 87.3% to 115.5%.

The established method was applied to determine fourteen metabolites in the hippocampus. The relative standard deviation (RSD) of quality control (QC) samples was less than 15% during sample runs. The concentrations of these metabolites fell within their respective linear ranges (Table S10). These results indicated that repeated exposure to soman significantly increased the levels of all twelve metabolites in the hippocampus and two metabolites were not quantified in the hippocampal tissue due to their concentrations being too low (Table S11).

## 4. Discussion

### 4.1. Calcium Dysregulation

The significant upregulation of calcium/calmodulin-dependent protein kinase II (CAMK2) observed in our study (Figure 6) provides compelling evidence for calcium-mediated neurotoxicity in soman-induced hippocampal injury. The activation of CAMK2 can trigger downstream signaling cascades, which in turn promote mitochondrial dysfunction and oxidative stress, ultimately leading to excitotoxic neuronal death [19,20]. Our findings are consistent with those of previous research showing that organophosphorus compounds induce sustained calcium influx that participates in neuropathological processes [21,22,23]. The observed increase in CAMK2 expression establishes a direct molecular link between soman exposure and calcium dysregulation in hippocampal neurons.

Concurrently, the downregulation of calcium-buffering proteins (calbindin D28k, calretinin, and parvalbumin) represents a maladaptive response to chronic calcium overload. These proteins normally function as endogenous neuroprotectants by regulating intracellular calcium homeostasis. Their diminished expression creates a permissive environment for calcium-dependent proteases and phosphatases to initiate apoptotic pathways, ultimately contributing to the neuronal depletion observed in hippocampal CA1 regions [24]. This calcium dysregulation cascade explains the spatial learning deficits demonstrated in Morris water maze tests, as calcium signaling is fundamental to synaptic plasticity and memory formation [25].

### 4.2. Amino Acid Catabolic Disruption

**Figure 7:**
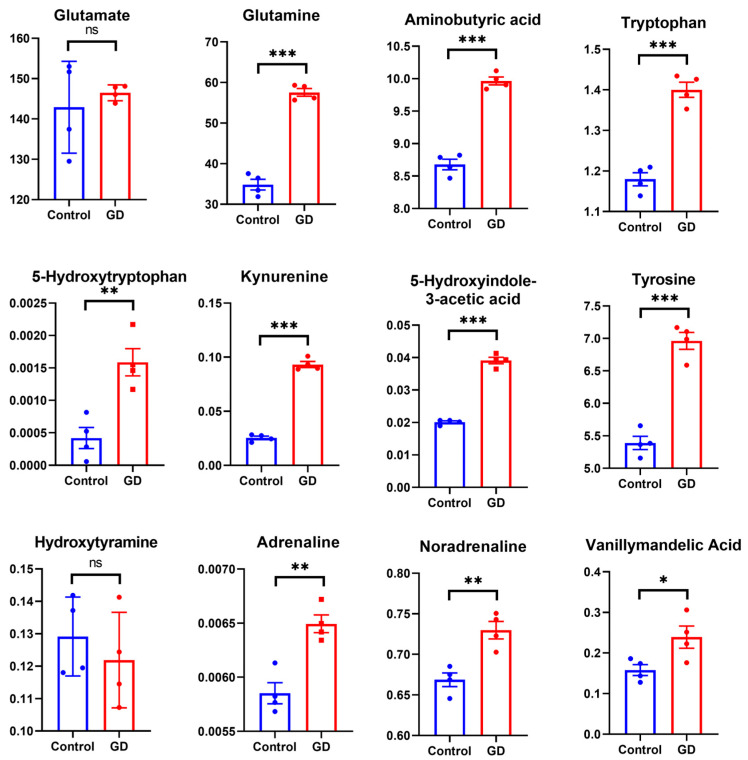
The results of neurotransmitters levels determined by LC–MS/MS in the guinea pig hippocampus following soman exposure. Data are presented as mean ± SEM. *n* = 4 for each treatment; ns indicated *p*-value > 0.05; * indicated *p*-value < 0.05, ** indicated *p*-value < 0.01, *** indicated *p*-value < 0.005 compared with control group. The Y-axis of all bar charts represents concentration (μg/mL). Data were analyzed using two-way analysis of variance and independent and paired-samples Student’s *t*-test.

Disorders of amino acid metabolism adversely affect brain energy metabolism and lead to the accumulation of amino acids or their metabolites, which are likely toxic to the nervous system [26]. Aberrant expressed ACAT2, ALDH, and MAOB were enriched in tryptophan metabolism suggesting that soman potential induces disrupted tryptophan metabolism. The tryptophan, 5-hydroxytryptophan, kynurenine, and 5-hydroxyindole-3-acetic acid (Figure 7), key intermediate metabolites in tryptophan metabolism, were significant elevated following soman exposure. Elevated kynurenine promotes excitotoxicity, potentially suppressing neuroprotective metabolites through NMDA receptor activation while simultaneously increasing oxidative stress [27].

In the alanine, aspartate, and glutamate metabolism, ABAT and ALDH5A1 were downregulated, suggesting that soman induces the impairment of alanine, aspartate, and glutamate metabolism. Glutamine and GABA (Figure 7), key intermediate metabolites in alanine, aspartate, and glutamate metabolism, were significant elevated following soman exposure relative to the control group. This disruption likely contributes to the neuronal hyperexcitability observed following soman exposure [28].

Aberrant expressed TH and MAOB were enriched in tyrosine metabolism. In this study, TH was downregulated, and MAOB was upregulated, suggesting that soman induces disrupted tyrosine metabolism. The L-tyrosine, hydroxytyramine, levodopa, adrenaline, noradrenaline, and vanillymandelic acid (Figure 7), key intermediate metabolites in tyrosine metabolism, were significant elevated following soman exposure suggesting compensatory mechanisms attempting to maintain neurotransmission amid cholinergic crisis. However, chronic elevation promotes oxidative damage through catecholamine autoxidation [29].

### 4.3. Glyoxylate and Dicarboxylate Metabolism Disruption

Aberrant expressed HYI, BPGM, and PGK1 were enriched in tyrosine metabolism, representing a novel finding that implicates disruption of the glyoxylate and dicarboxylate metabolism pathway in soman neurotoxicity. HYI catalyzes the interconversion of hydroxypyruvate and glyceraldehyde, bridging glycolysis with gluconeogenesis and glycine metabolism. The reduction in HYI expression (validated by ELISA) likely contributes to the accumulation of toxic metabolic intermediates, which may explain the selective neuronal vulnerability observed in our histopathological analysis [30]. Additionally, it disrupts photorespiration-derived glycine synthesis and shunts glycolytic intermediates away from energy production. This observation is reinforced by the parallel downregulation of BPGM and PGK1, indicating broad impairment of glycolytic flux [31].

Our findings regarding calcium dysregulation and acetylcholinesterase inhibition are consistent with the established mechanisms of organophosphorus compound toxicity [21]. However, the comprehensive mapping of metabolic disruptions represents a significant advancement in understanding the subacute and chronic consequences of soman exposure. While previous studies have documented mitochondrial dysfunction following nerve agent exposure, our identification of specific pathway alterations provides mechanistic insights into the metabolic basis of neuronal vulnerability [32].

### 4.4. Limitations of This Study

This study has several limitations that should be considered. Firstly, our investigation was primarily focused on the histopathological and functional alterations within the hippocampus following soman exposure. While the hippocampus is a primary target and critically involved in the cognitive deficits associated with organophosphate poisoning, we acknowledge that the organophosphate nerve agent affects other brain regions, inducing widespread neurotoxicity, such as delayed neuropathy and epileptic seizures [33,34,35]. The contributions of these extra-hippocampal effects to the overall pathophysiology and behavioral outcomes measured in our study were not explored and represent an important avenue for future research. A more comprehensive, multi-organ approach in subsequent studies will be essential to fully elucidate the complex mechanisms of soman-induced toxicity.

## 5. Conclusions

**Figure 8:**
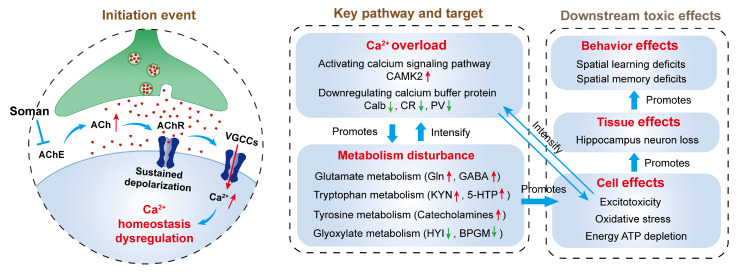
Schematic diagram of mechanisms of metabolic dysregulation in soman neurotoxicity.

This study establishes that soman-induced hippocampal damage extends beyond acute cholinergic crisis to involve multi-pathway metabolic dysregulation. Through calcium-mediated disruption of interconnected metabolic networks—glyoxylate/dicarboxylate, tryptophan, tyrosine, and glutamate pathways—soman triggers a self-sustaining cycle of energy failure, excitotoxicity, and oxidative stress that culminates in neuronal loss and cognitive impairment. The key molecular events include CAMK2 upregulation, HYI downregulation, and the accumulation of neurotoxic metabolites (kynurenine, catecholamines, and glutamine). These findings reposition organophosphate neurotoxicity as a metabolic disorder with secondary excitotoxic components, suggesting that therapeutic strategies targeting metabolic recovery—calcium buffering, alternative energy substrates, and kynurenine pathway inhibition—may prove more effective than solely addressing cholinergic symptoms (Figure 8). Future work should validate these targets in chronic recovery models and assess their translational potential for mitigating the long-term neuropsychiatric sequelae of OPNA exposure.

## Figures and Tables

**Table 1:** The top five differentially expressed proteins in hippocampus after subacute soman exposure.

Protein Name	Accession	Abbreviations	Pathway	Fold Change	*p* Value
Calcium/calmodulin-dependent protein kinase	A0A286XFT7	CAMK2	Calcium signal pathway	1.963	0.000001
Tyrosine 3-hydroxylase	H0VLS4	TH	Tyrosine metabolism	0.357	0.001205
Sphingomyelin phosphodiesterase 3	H0VT86	SMPD3	Glycerophospholipid metabolis	56.657	0.001873
Phosphoglycerate mutase	H0UTW8	BPGM	Glycolysis/Gluconeogenesis	0.021	0.000579
Putative hydroxypyruvate isomerase	H0V732	HYI	Glyoxylate and dicarboxylate metabolism	0.4	0.000159

## Data Availability

The data presented in this research are available in this article and Supplementary Materials.

## Supplementary Materials

The following supporting information can be downloaded at: https://www.mdpi.com/article/10.3390/toxics13090766/s1, Figure S1: GO functional annotation for differential protein after subacute soman exposure in category of “BP”; Figure S2: KEGG annotation for differential protein after subacute soman exposure in category of “metabolism”; Figure S3: The total ion chromatograms of those standards, samples, and blank matrix; Table S1: The information of differential proteins annotating in the first category of “metabolism”; Table S2: Differentially expressed proteins enriched into calcium signal transduction and transport in hippocampus after 0.2 LD50 soman exposure; Table S3: Differentially expressed proteins enriched into amino acid metabolism in hippocampus after 0.2 LD50 soman exposure; Table S4: Differentially expressed proteins enriched into lipid metabolism in hippocampus after 0.2 LD50 soman exposure; Table S5: Differentially expressed proteins enriched into carbohydrate metabolism in hippocampus after 0.2 LD50 soman exposure; Table S6: Specific analyte information: ion for metabolites in specific amino acid metabolic pathways; Table S7: Details of concentration levels for analytes determination; Table S8: The specific transitions used for analyte quantification; Table S9: Details of linear equation for analyte quantification; Table S10: Results of methodology investigation for analytes; Table S11. Results of determination for analytes.
