# Supplementary material for: Proteomics Reveals Mechanisms of Metabolic Dysregulation in Soman Neurotoxicity
Source: Toxics. 2025 Sep 10;13(9):766. doi: 10.3390/toxics13090766 (PMC12474405; doi:10.3390/toxics13090766)
Supplement: Supplementary file 1 [file toxics-13-00766-s001.zip › toxics-3810559-supplementary.pdf]

# Supporting information

## Proteomic mapping of metabolic dysregulation in soman neurotoxicity

*Xing-Xing Zong, Qian Jin, Tong Shi, Ruihua Zhang, Jingjing Shi, Chen Wang* and Liqin Li**

State Key Laboratory of NBC Protection for Civilian, Beijing, 102205, PR China.
E-mail: wangchenpla@163.com; llq969696@126.com

## Table and figure legends:

**Table S1.** The information of differential proteins annotating in the firt category of "Metabolism".

**Table S2.** Differentially expressed proteins enriched into calcium signal transduction and transport in hippocampus after 0.2 $LD_{50}$ soman exposure.

**Table S3.** Differentially expressed proteins enriched into amino acid metabolish in hippocampus after 0.2 $LD_{50}$ soman exposure.

**Table S4.** Differentially expressed proteins enriched into lipid metabolism in hippocampus after 0.2 $LD_{50}$ soman exposure.

**Table S5.** Differentially expressed proteins enriched into carbohydrate metabolism in hippocampus after 0.2 $LD_{50}$ soman exposure.

**Table S6.** Specific analytes informationion for metabolites in specific amino acid metabolic pathways.

**Table S7.** Detailed of concentration levels for analytes determination.

**Table S8.** The specific transitions used for analytes quantification

**Table S9.** Detailed of linear equation for analytes quantification

**Table S10.** Results of methodology investigation for analytes

**Table S11.** Results of determination for analytes

**Figure S1.** GO functional annotation for differential protein after subacute soman exposure in category of "BP".

**Figure S2.** KEGG annotation for differential protein after subacute soman exposure in category of "metabolism".

**Figure S3.** The total ion chromatograms of those standards, samples and blank matrix.

**Table S1.** The information of differential proteins annotating in the first category of "Metabolism".

| Accession | Gene name | Ko id | Description | Unique Peptides | Coverage | MW [kDa] | FDR Confidence | regulate |
|---|---|---|---|---|---|---|---|---|
| A0A286XFT7 | CAMK2A | K04515 | Calcium/calmodulin-dependent protein kinase | 18 | 58 | 54.1 | High | up |
| H0VS40 | PRKCA | K02677 | Protein kinase C alpha typ | 16 | 45 | 68.7 | High | up |
| A0A286XJB7 | CAMK2G | K04515 | Calcium/calmodulin-dependent protein kinase | 3 | 48 | 58.2 | High | up |
| A0A286Y2C2 | PPP3R | K06268 | Protein phosphatase 3 regulatory subunit B | 11 | 67 | 19.2 | High | up |
| B2ZPE9 | CALMk4 | K05869 | Calmodulin | 7 | 86 | 16.8 | High | up |
| H0V9W6 | ASPH | K00476 | Aspartate beta-hydroxylase | 9 | 19 | 72.9 | High | up |
| H0UUB7 | CB-D28k | K14757 | Calbindin D28k | 15 | 54 | 31.7 | High | down |
| H0VGS0 | CR | - | Calretinin | 17 | 74 | 31.3 | High | down |
| H0W915 | PV | - | Parvalbumin | 4 | 55 | 12.2 | High | down |
| H0UX23 | CKM | K00933 | Creatine kinase | 19 | 56 | 47.1 | High | down |
| H0UWR3 | ACAT2 | K00626 | Acetyl-CoA acetyltransferase | 19 | 72 | 41.2 | High | down |
| H0VCS7 | GOT1 | K14454 | Aspartate aminotransferase | 25 | 70 | 46.2 | High | down |
| A0A286XJ12 | ABAT | K13524 | (S)-3-amino-2-methylpropionate transaminase | 31 | 77 | 56.4 | High | down |
| H0UTW8 | BPGM | K01837 | Phosphoglycerate mutase | 8 | 38 | 29.9 | High | down |
| A0A286XYN2 | MDH1 | K00025 | Malate dehydrogenase | 0 | 0 | 0 | High | down |
| A0A286XTR9 | ADH5 | K00121 | S-(hydroxymethyl)glutathione dehydrogenase | 10 | 43 | 38.9 | High | down |
| H0W439 | HIBADH | K00020 | 3-hydroxyisobutyrate dehydrogenase | 9 | 53 | 31.3 | High | down |
| A0A286XLF3 | ALDH5A1 | K00139 | Succinate-semialdehyde dehydrogenase | 0 | 0 | 0 | High | down |
| H0VM89 | BCAT2 | K00826 | Branched-chain-amino-acid aminotransferase | 13 | 41 | 44.2 | High | down |
| H0VRK8 | MMUT | K01847 | Methylmalonyl-CoA isomerase | 22 | 45 | 82.8 | High | down |
| A0A286XDA0 | GAMT | K00542 | Guanidinoacetate N-methyltransferase | 8 | 61 | 26.5 | High | down |
| H0UV57 | MAOB | K00274 | Amine oxidase | 3 | 54 | 58.3 | High | up |
| H0VLS4 | TH | K00501 | Tyrosine 3-hydroxylase | 15 | 52 | 55.9 | High | down |
| H0W0T5 | ASPG | K13278 | Asparaginase | 32 | 45 | 60.7 | High | up |
| H0VEQ3 | PADI2 | K01481 | Protein-arginine deiminase | 23 | 42 | 75.4 | High | down |
| A0A286XRU5 | PGK1 | K00927 | Phosphoglycerate kinase | 30 | 80 | 44.6 | High | down |
| H0VQ67 | PGM2L1 | K11809 | Phosphoglucomutase 2 like 1 | 28 | 54 | 70.2 | High | down |
| H0V732 | HYI | K01816 | Putative hydroxypyruvate isomerase | 7 | 42 | 29.3 | High | down |
| H0V0W5 | ACSS3 | K01908 | Propionate--CoA ligase | 13 | 26 | 75.7 | High | down |
| H0VZZ5 | PGM1 | K01835 | Phosphoglucomutase 1 | 22 | 49 | 63.6 | High | down |
| H0VRK8 | MMUT | K01847 | Methylmalonyl-CoA isomerase | 22 | 45 | 82.8 | High | down |
| H0V7J0 | HEXB | K12373 | Beta-N-acetylhexosaminidase | 12 | 28 | 50.7 | High | down |
| A0A286XW41 | DGKG | K00901 | Diacylglycerol kinase | 21 | 36 | 89.5 | High | down |
| A0A286XL13 | MGLL | K01054 | Monoglyceride lipase | 0 | 0 | 0 | High | down |
| A0A286X9H4 | DGKZ | K00901 | Diacylglycerol kinase | 1 | 30 | 104.9 | High | up |
| A0A286XZ54 | ACOT1_2_4 | K01068 | Uncharacterized protein | 5 | 35 | 50.3 | High | up |
| A0A286XYK9 | LYPLA2 | K06130 | Lysophospholipase 2 | 28 | 64 | 24.7 | High | up |
| H0VT86 | SMPD3 | K12352 | Sphingomyelin phosphodiesterase 3 | 32 | 55 | 71.2 | High | up |

**Table S2** Differentially expressed proteins enriched into calcium signal transduction and transport in hippocampus after 0.2 $LD_{50}$ soman exposure.

| **Pathway** | **Accession** | **Protein name** | **Abbreviations** | **Fold change** | **P value** |
|---|---|---|---|---|---|
| **Calcium signal** | A0A286XFT7 | Calcium/calmodulin-dependent protein kinase | CAMK2A | 1.963 | 0.000001036 |
| | A0A286XJB7 | Non-specific protein-tyrosine kinase | CAMK2G | 1.224 | 0.001285 |
| | H0VS40 | Protein kinase C alpha type | PRKCA | 1.416 | 0.0003979 |
| | A0A286Y2C2 | Protein phosphatase 3 regulatory subunit B | PPP3R | 1.486 | 0.0001689 |
| | B2ZPE9 | Calmodulin | CALM | 1.261 | 0.0009018 |
| **Calcium buffering** | H0UUB7 | Calbindin D28k | CB-D28k | 0.634 | 0.0001048 |
| | H0VGS0 | Calretinin | CR | 0.61 | 0.00006879 |
| | H0W915 | Parvalbumin | PV | 0.587 | 0.0004314 |

**Table S3** Differentially expressed proteins enriched into amino acid metabolish in hippocampus after 0.2 $LD_{50}$ soman exposure.

| Pathway | Accession | Protein name | Abbreviations | Fold change | P value |
|---|---|---|---|---|---|
| **Valine, leucine and isoleucine degradation** | H0VM89 | Branched-chain-amino-acid aminotransferase | BCAT2 | 0.746 | 0.000256 |
| | H0W439 | 3-hydroxyisobutyrate dehydrogenase | HIBADH | 0.775 | 0.00065 |
| | H0VRK8 | Methylmalonyl-CoA isomerase | MMUT | 0.719 | 0.000321 |
| | H0UWR3 | Acetyl-CoA acetyltransferase | ACAT2 | 0.694 | 0.000728 |
| **tryptophan metabolism** | A0A286XPF7 | aldehyde dehydrogenase | ALDH | 1.67 | 0.000145 |
| | H0UV57 | Amine oxidase | MAOB | 1.299 | 0.000537 |
| | H0UWR3 | Acetyl-CoA acetyltransferase | ACAT2 | 0.694 | 0.000728 |
| **Cysteine and methionine metabolism** | A0A286XYN2 | Malate dehydrogenase | MDH1 | 0.735 | 0.000501 |
| | H0VCS7 | Aspartate aminotransferase | GOT1 | 0.82 | 0.000716 |
| **Alanine, aspartate and glutamate metabolism** | A0A286XJ12 | (S)-3-amino-2-methylpropionate transaminase | ABAT | 0.658 | 0.000105 |
| | H0VCS7 | Aspartate aminotransferase | GOT1 | 0.82 | 0.000716 |
| | A0A286XLF3 | Succinate-semialdehyde dehydrogenase | ALDH5A1 | 0.588 | 0.001554 |
| **Tyrosine metabolism** | H0VCS7 | Aspartate aminotransferase | GOT1 | 0.82 | 0.000716 |
| | H0VLS4 | Tyrosine 3-hydroxylase | TH | 0.357 | 0.001205 |
| | A0A286XTR9 | S-(hydroxymethyl)glutathione dehydrogenase | ADH5 | 0.746 | 0.000292 |
| | H0UV57 | Amine oxidase | MAOB | 1.299 | 0.000537 |

**Table S4** Differentially expressed proteins enriched into lipid metabolism in hippocampus after 0.2 $LD_{50}$ soman exposure.

| Pathway | Accession | Protein name | Abbreviations | Fold change | P value |
|---|---|---|---|---|---|
| Glycerophospholipid metabolis | A0A286XW41 | Diacylglycerol kinase | DGKG | 1.919 | 0.002956 |
| | A0A286XL13 | Monoglyceride lipase | MGLL | 1.26 | 0.00007549 |
| | H0VT86 | Sphingomyelin phosphodiesterase 3 | SMPD3 | 56.657 | 0.001873 |
| | A0A286X9H4 | Diacylglycerol kinase | DGKZ | 1.588 | 0.0001113 |
| | A0A286XYK9 | Lysophospholipase 2 | LYPLA2 | 1.205 | 0.02897 |
| Fatty acid degradation | H0UWR3 | Acetyl-CoA acetyltransferase 2 | ACAT2 | 0.694 | 0.000728 |
| | A0A286XTR9 | S-(hydroxymethyl)glutathione dehydrogenase | ADH5 | 0.746 | 0.000292 |

**Table S5** Differentially expressed proteins enriched into carbohydrate metabolism in hippocampus after 0.2 $LD_{50}$ soman exposure.

| Pathway | Accession | Protein name | Abbreviations | Fold change | P value |
|---|---|---|---|---|---|
| Glycolysis/ Gluconeogenesis | A0A286XRU5 | Phosphoglycerate kinase | PGK1 | 0.769 | 0.00004676 |
| | H0UTW8 | Phosphoglycerate mutase | BPGM | 0.021 | 0.0005792 |
| | H0VZZ5 | Phosphoglucomutase 1 | PGM1 | 0.802 | 0.003111 |
| | H0VQ67 | Phosphoglucomutase 2 like 1 | PGM2L1 | 0.748 | 0.001945 |
| Glyoxylate and dicarboxylate metabolism | H0VRK8 | Methylmalonyl-CoA isomerase | MMUT | 0.717 | 0.0003211 |
| | H0UWR3 | Acetyl-CoA acetyltransferase 2 | ACAT2 | 0.694 | 0.0007278 |
| | H0V732 | Putative hydroxypyruvate isomerase | HYI | 0.4 | 0.0001591 |
| | A0A286XYN2 | Malate dehydrogenase | MDH1 | 0.732 | 0.000500693 |

**Table S6.** Specific analytes informationion for metabolites in specific amino acid metabolic pathways.

| No | Analyte | Abbreviation | CAS |
|---|---|---|---|
| 1 | Glutamate | Glu | 56-86-0 |
| 2 | L-Glutamine | Gln | 56-85-9 |
| 3 | 4-Aminobutyric acid | GABA | 56-12-2 |
| 4 | L-Tryptophan | Trp | 73-22-3 |
| 5 | Serotonin | 5-HT | 153-98-0 |
| 6 | 5-Hydroxytryptophan | 5-HTP | 4350-09-8 |
| 7 | L-Kynurenine | Kyn | 343-65-7 |
| 8 | 5-Hydroxyindole-3-acetic acid | 5-HIAA | 54-16-0 |
| 9 | L-Tyrosine | Tyr | 60-18-4 |
| 10 | Hydroxytyramine | DA | 62-31-7 |
| 11 | Levodopa | DOPA | 59-92-7 |
| 12 | Adrenaline | E | 329-63-5 |
| 13 | Noradrenaline | NE | 55-27-6 |
| 14 | Vanillymandelic Acid | VMA | 55-10-7 |

**Table S7.** Detailed of concentration levels for analytes determination.

| Analyte | 1 | 2 | 3 | 4 | 5 | 6 | 7 | 8 | 9 | 10 | 11 | 12 |
|---|---|---|---|---|---|---|---|---|---|---|---|---|
| Glu | - | 1000 | 500 | 400 | 200 | 100 | 80 | 40 | 20 | 10 | 4 | 2 |
| Gln | - | 500 | 250 | 200 | 100 | 50 | 40 | 20 | 10 | 5 | 2 | 1 |
| GABA | 1000 | 500 | 250 | 200 | 100 | 50 | 40 | 20 | 10 | 5 | 2 | 1 |
| Trp | - | 250 | 125 | 100 | 50 | 25 | 20 | 10 | 5 | 2.5 | 1 | 0.5 |
| 5-HT | 1000 | 500 | 250 | 200 | 100 | 50 | 40 | 20 | 10 | 5 | 2 | 1 |
| 5-HTP | 500 | 250 | 125 | 100 | 50 | 25 | 20 | 10 | 5 | 2.5 | 1 | 0.5 |
| Kyn | 2000 | 1000 | 500 | 400 | 200 | 100 | 80 | 40 | 20 | 10 | 4 | 2 |
| 5-HIAA | - | 500 | 250 | 200 | 100 | 50 | 40 | 20 | 10 | 5 | 2 | 1 |
| Tyr | 2500 | 1250 | 625 | 500 | 250 | 125 | 100 | 50 | 25 | 12.5 | 5 | 2.5 |
| DA | - | 250 | 125 | 100 | 50 | 25 | 20 | 10 | 5 | 2.5 | 1 | 0.5 |
| DOPA | - | 1250 | 625 | 500 | 250 | 125 | 100 | 50 | 25 | 12.5 | 5 | 2.5 |
| E | 250 | 125 | 62.5 | 50 | 25 | 12.5 | 10 | 5 | 2.5 | 1.25 | 0.5 | 0.25 |
| NE | - | - | 250 | 200 | 100 | 50 | 40 | 20 | 10 | 5 | 2 | 1 |
| VMA | 5000 | 2500 | 1250 | 1000 | 500 | 250 | 200 | 100 | 50 | 25 | - | - |

**Table S8.** The specific transitions used for analytes quantification

| Analyte | Precursor | Monitoring ion | DP | EP | CE | CXP |
|---|---|---|---|---|---|---|
| Glu | 148.086 | 84.2 | 36 | 10 | 23 | 6 |
| Gln | 147.096 | 130.0 | 41 | 10 | 15 | 12 |
| GABA | 104.051 | 87.0 | 31 | 10 | 15 | 6 |
| Trp | 205.106 | 188.3 | 41 | 10 | 15 | 18 |
| 5-HT | 177.173 | 160.1 | 36 | 10 | 15 | 16 |
| 5-HTP | 221.106 | 204.2 | 46 | 10 | 17 | 6 |
| Kyn | 209.089 | 192.2 | 46 | 10 | 13 | 4 |
| 5-HIAA | 192.048 | 146.0 | 56 | 10 | 21 | 14 |
| Tyr | 182.137 | 136.1 | 31 | 10 | 19 | 12 |
| DA | 154.142 | 137.1 | 36 | 10 | 15 | 12 |
| DOPA | 198.113 | 152.0 | 41 | 10 | 19 | 14 |
| E | 184.078 | 166.2 | 31 | 10 | 15 | 32 |
| NE | 170.117 | 152.0 | 31 | 10 | 13 | 14 |
| VMA | 221.059 | 203.0 | 41 | 10 | 11 | 6 |
| Phe-1-13C | 167.085 | 120.000 | 56 | 10 | 19 | 10 |

**Table S9.** Detailed of linear equation for analytes quantification

| Analyte | RT (min) | Linear equation | $R^2$ | linear range (ng/mL) | LOQ (ng/mL) | RSD (%) |
|---|---|---|---|---|---|---|
| Glu | 4.41 | y=14640x + 7677 | 0.9921 | 2~1000 | 2 | 0.75 |
| Gln | 4.32 | y=19290x + 19090 | 0.9946 | 1~500 | 1 | 1.02 |
| GABA | 4.25 | y=13810x + 4019 | 0.9962 | 1~1000 | 1 | 2.95 |
| Trp | 10.95 | y=42590x + 44210 | 0.9916 | 0.5~250 | 0.5 | 2.98 |
| 5-HT | 6.47 | y=37180x + 4292 | 0.9917 | 1~1000 | 1 | NA |
| 5-HTP | 7.29 | y=28460x + 130.3 | 0.999 | 0.5~500 | 0.5 | NA |
| Kyn | 8.54 | y=8609x + 1531 | 0.9977 | 2~2000 | 2 | 4.02 |
| 5-HIAA | 11.09 | y=24220x + 3340 | 0.9956 | 1~500 | 1 | 5.77 |
| Tyr | 6.24 | y=11160x + 8450 | 0.996 | 2.5~2500 | 2.5 | 1.92 |
| DA | 4.85 | y=24780x - 2284 | 0.9934 | 0.5~250 | 0.5 | NA |
| DOPA | 5.39 | y=17580x + 5239 | 0.9954 | 2.5~1250 | 2.5 | NA |
| E | 4.4 | y=51440x - 2682 | 0.9974 | 0.25~250 | 0.25 | NA |
| NE | 4.29 | y=18110x - 1202 | 0.9953 | 1~250 | 1 | 1.12 |
| VMA | 8.35 | y=1060x + 948.3 | 0.9993 | 25~5000 | 25 | NA |

**Table S10.** Results of methodology investigation for analytes

| Analyte | Concentration (ng/mL) | Precision Intra-day (%) | Precision Inter-day (%) | Recovery (%) | Repeatability (%) |
|---|---|---|---|---|---|
| GABA | 10 | 2.44 | 6.23 | 94.20 | 7.05 |
| | 50 | 3.29 | 5.77 | 100.13 | |
| | 250 | 7.89 | 6.26 | 86.40 | |
| Gln | 5 | 2.15 | 6.53 | 97.48 | 7.57 |
| | 25 | 6.97 | 6.69 | 93.83 | |
| | 10 | 4.80 | 8.12 | 98.75 | |
| | 50 | 4.43 | 6.69 | 98.68 | |
| | 250 | 6.84 | 5.59 | 94.38 | |
| Glu | 20 | 3.60 | 14.59 | 96.75 | 8.14 |
| | 100 | 4.58 | 6.21 | 99.35 | |
| | 500 | 9.03 | 9.97 | 95.07 | |
| DA | 5 | 5.59 | 7.81 | 98.04 | 2.19 |
| | 25 | 4.42 | 6.01 | 98.43 | |
| | 125 | 8.13 | 6.56 | 89.85 | |
| NE | 5 | 4.30 | 9.12 | 96.93 | 5.76 |
| | 25 | 8.32 | 10.87 | 92.93 | |
| | 10 | 5.36 | 9.37 | 91.25 | |
| | 50 | 5.31 | 6.05 | 102.29 | |
| | 250 | 8.37 | 8.01 | 92.75 | |
| 5-HT | 10 | 6.74 | 6.77 | 91.38 | 2.37 |
| | 50 | 3.98 | 6.41 | 89.47 | |
| | 250 | 8.41 | 9.82 | 86.3 | |
| Tyr | 25 | 9.24 | 7.78 | 105.5 | 9.30 |
| | 125 | 4.86 | 6.91 | 103.1 | |
| | 625 | 7.64 | 6.92 | 92.22 | |
| E | 2.5 | 6.79 | 10.21 | 96.05 | 11.85 |
| | 12.5 | 5.71 | 8.05 | 93.66 | |
| | 62.5 | 8.20 | 7.51 | 89.8 | |
| 5-HIAA | 50 | 4.93 | 8.20 | 90.26 | 7.12 |
| | 250 | 9.67 | 11.62 | 89.48 | |
| | 10 | 4.68 | 14.91 | 98.25 | |
| | 50 | 4.43 | 6.87 | 97.51 | |
| | 250 | 8.25 | 14.64 | 91.83 | |
| DOPA | 25 | 3.98 | 7.33 | 94.68 | 6.68 |
| | 125 | 2.95 | 9.63 | 96.00 | |
| | 625 | 9.10 | 11.51 | 93.8 | |
| Trp | 5 | 3.79 | 5.51 | 93.75 | 8.30 |
| | 25 | 4.87 | 6.36 | 101.79 | |

| | | | | | |
|---|---|---|---|---|---|
| | 125 | 9.38 | 7.86 | 91.50 | |
| | 100 | 3.06 | 5.93 | 89.36 | |
| | 500 | 8.36 | 5.81 | 86.08 | |
| | 2 | 10.46 | 10.16 | 94.50 | |
| Kyn | 10 | 7.89 | 9.40 | 91.14 | 5.78 |
| | 50 | 7.04 | 5.21 | 86.56 | |
| | 50 | 3.27 | 4.91 | 97.44 | |
| VMA | 250 | 5.48 | 5.96 | 93.4 | ND |
| | 1250 | 9.10 | 7.72 | 89.5 | |
| | 5 | 6.82 | 11.72 | 88.58 | |
| | 25 | 6.59 | 7.82 | 90.20 | |
| 5-HTP | 125 | 8.61 | 6.86 | 85.54 | 9.13 |
| | 5 | 5.37 | 8.03 | 87.08 | |
| | 25 | 9.45 | 9.32 | 85.63 | |

**Table S11.** Results of determination for analytes

| Analyte | Control | | | | GD | | | |
|---|---|---|---|---|---|---|---|---|
| | 1 | 2 | 3 | 4 | 1 | 2 | 3 | 4 |
| Glu | 137.434 | 153.0128607 | 151.6737 | 129.5135 | 143.8747 | 146.0386 | 148.1189 | 147.8632 |
| Gln | 33.44865 | 37.53536547 | 36.45219 | 31.87353 | 55.66936 | 56.16877 | 59.33058 | 59.06445 |
| GABA | 8.635909 | 8.821330775 | 8.467613 | 8.787964 | 9.994446 | 9.841661 | 9.905345 | 10.12121 |
| Trp | 1.138852 | 1.201249589 | 1.209508 | 1.169096 | 1.434214 | 1.352747 | 1.387809 | 1.426023 |
| 5-HT | 0 | 0 | 0 | 0 | 0 | 0 | 0 | 0 |
| 5-HTP | 0.000525 | 5.67323E-05 | 0.000816 | 0.000284 | 0.001549 | 0.002169 | 0.001461 | 0.00117 |
| Kyn | 0.021345 | 0.028328726 | 0.024607 | 0.027576 | 0.089674 | 0.088921 | 0.093437 | 0.100964 |
| 5-HIAA | 0.020185 | 0.020660611 | 0.020661 | 0.018981 | 0.036476 | 0.039344 | 0.039329 | 0.041306 |
| Tyr | 5.157629 | 5.654241935 | 5.366113 | 5.384823 | 7.105306 | 6.589242 | 7.167661 | 6.988145 |
| DA | 0.128484 | 0.130227603 | 0.192697 | 0.116862 | 0.171196 | 0.088969 | 0.154925 | 0.211874 |
| DOPA | 0 | 0 | 0 | 0 | 0 | 0 | 0 | 0 |
| E | 0.005823 | 0.005767418 | 0.005683 | 0.006131 | 0.006719 | 0.006495 | 0.006418 | 0.006341 |
| NE | 0.685294 | 0.675116289 | 0.668715 | 0.645597 | 0.702708 | 0.722904 | 0.743141 | 0.750516 |
| VMA | 0.239027 | 0.277928571 | 0.157269 | 0.227489 | 0.211995 | 0.127599 | 0.213313 | 0.30628 |

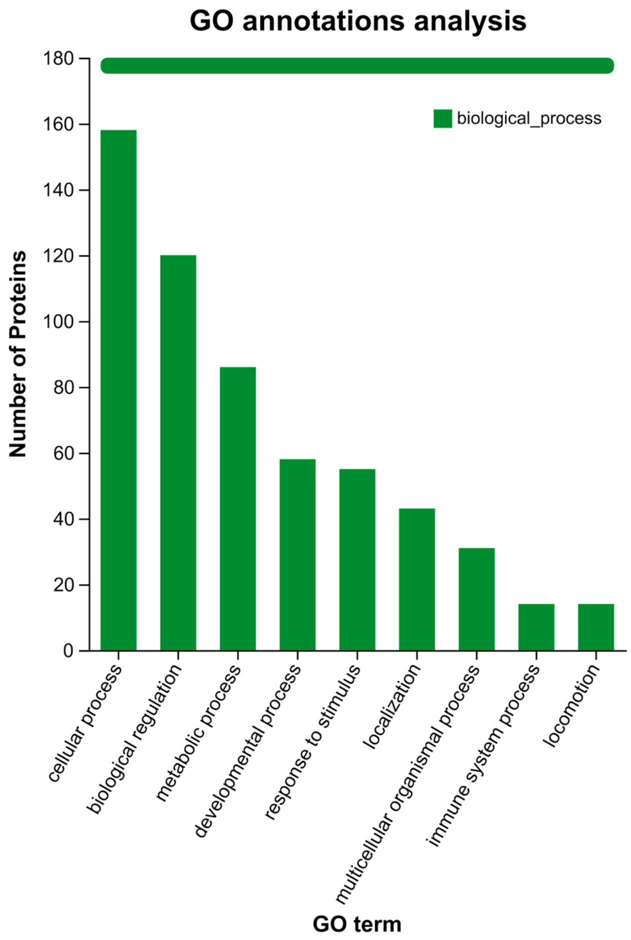


**Figure S1.** GO functional annotation for differential protein after subacute soman exposure in category of “BP”.

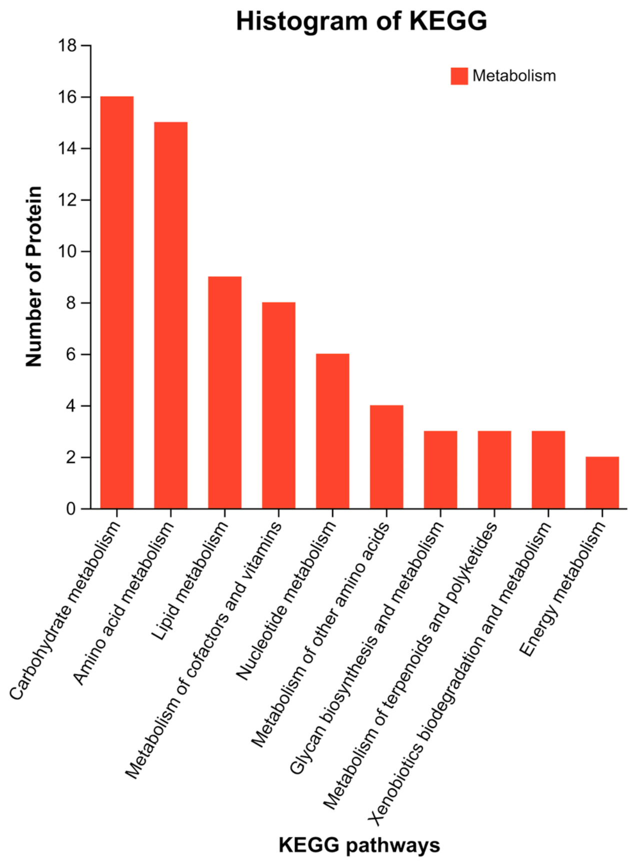


**Figure S2.** KEGG annotation for differential protein after subacute soman exposure in category of "metabolism".

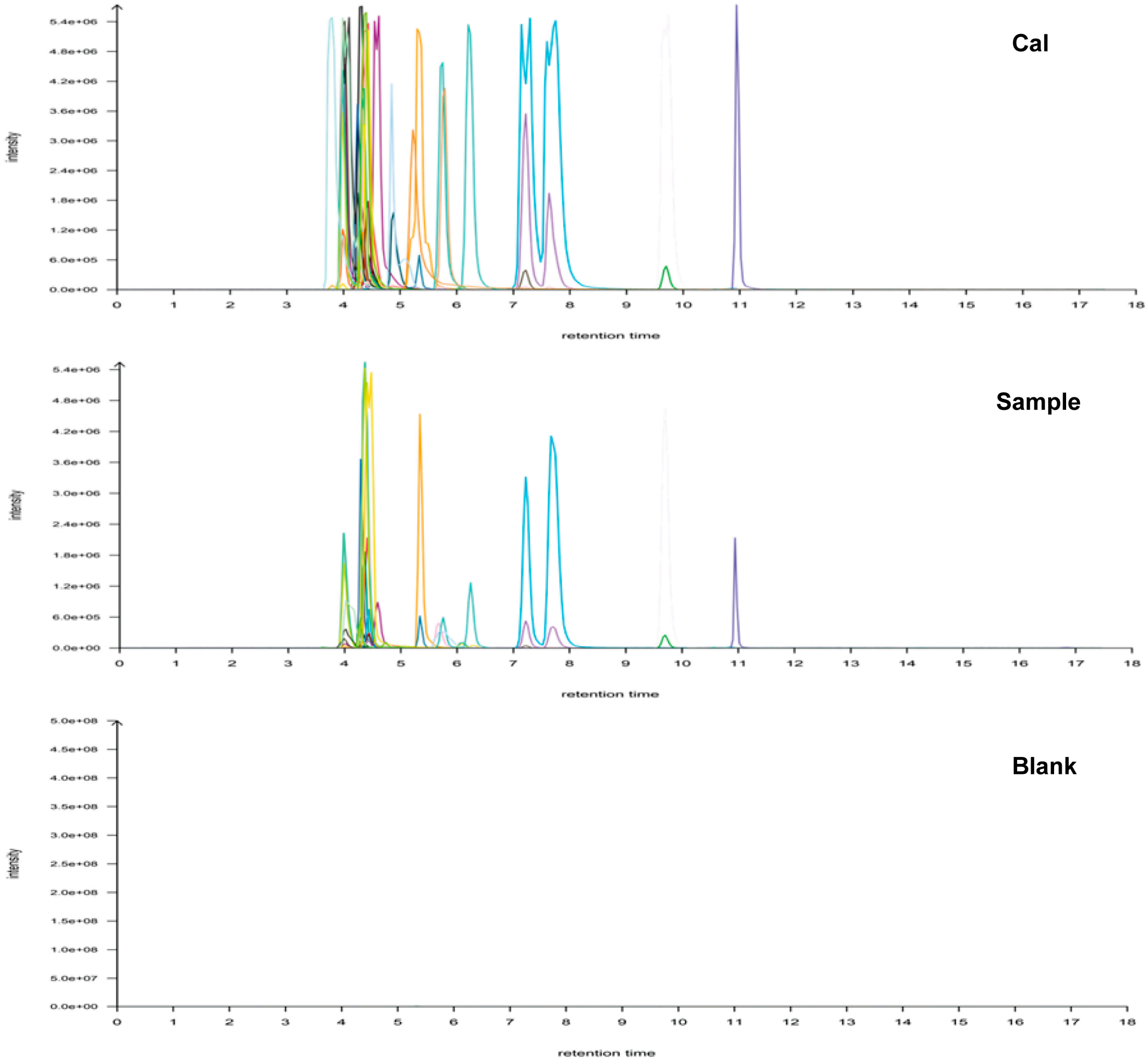


**Figure S3.** The total ion chromatograms of those standards, samples and blank matrix.
